# Key factors for successful gait acquisition in individuals with severe hemiplegic subacute stroke using a single-legged gait-training assistance robot: a retrospective cohort study

**DOI:** 10.1186/s12984-026-01961-4

**Published:** 2026-03-28

**Authors:** Tomoya Asano, Norihide Itoh, Tatsuyoshi Ikenoue, Ryo Okochi, Takuma Ii, Satoshi Hirano

**Affiliations:** 1 Rehabilitation Department, Okayama Rehabilitation Hospital, 503-1 Kurata, Naka, Okayama, 703-8265 Okayama Japan; 2 Faculty of Rehabilitation, School of Health Sciences, Fujita Health University, Toyoake, Japan; 3 Department of Rehabilitation Medicine, Saiseikai Moriyama Municipal Hospital, Moriyama, Japan; 4 Data Science and AI Innovation Research Promotion Center, Shiga University, Hikone, Japan; 5 Department of Rehabilitation Medicine, School of Medicine, Fujita Health University, Toyoake, Japan

**Keywords:** Stroke, Gait independence, Robotics, Rehabilitation, Walking

## Abstract

**Background:**

Robot-assisted gait training (RAGT) has been explored using various devices. Most studies have focused on bilateral-legged devices; however, few have evaluated single-legged devices. Therefore, we aimed to identify key factors associated with successful responders among individuals with severe hemiplegic subacute stroke who had ambulatory deficits and underwent gait training using a single-legged assistance robot.

**Methods:**

We retrospectively analyzed patients with stroke who received RAGT at Okayama Rehabilitation Hospital between February 2018 and September 2024. Eligible patients had severe hemiplegia after a first-time supratentorial stroke and required assistance for ambulation. Exclusion criteria included subarachnoid hemorrhage, concomitant neurological disease, or failure to complete 10 RAGT sessions. The primary outcome was achieving walking with supervision or better, rather than requiring physical assistance. Responders were defined as those who walked without physical assistance but required supervision. We explored factors affecting this outcome after 4 weeks of RAGT. Factor selection was performed using the Direct Linear Non-Gaussian Acyclic Model, followed by logistic regression to assess associations.

**Results:**

A total of 126 participants were included. Stroke Impairment Assessment Set (SIAS) scores (knee extension, ankle dorsiflexion, joint position sense, trunk verticality, and abdominal strength), Functional Independence Measure scores (problem solving and memory), time from onset to RAGT initiation, and initial walking independence were associated with achieving walking with supervision. Among these factors, days to RAGT initiation (odds ratio [OR] = 0.19), SIAS trunk verticality (OR = 7.79), and SIAS joint position sense (OR = 3.37) were independently associated with the outcome.

**Conclusions:**

Achieving walking with supervision was associated with earlier initiation of RAGT, trunk control, and lower-limb proprioception in patients with severe hemiplegia undergoing gait training with a single-legged assistance robot. These findings highlight key factors for selecting candidates for single-legged gait-training robots and may guide more effective rehabilitation strategies.

*Trial Registration* University Hospital Medical Information NetworkClinical Trials Registry UMIN000056551 January 4, 2025.

**Supplementary Information:**

The online version contains supplementary material available at 10.1186/s12984-026-01961-4.

## Background

Robot-assisted gait training (RAGT) combined with conventional rehabilitation improves walking independence more effectively in individuals with stroke than conventional rehabilitation alone [1]. These benefits are particularly evident in non-ambulatory individuals with stroke within 3 months after onset. Systematic reviews and clinical guidelines suggest that RAGT is more effective for individuals with severe impairments; however, clarifying the characteristics of those most likely to benefit from specific robotic devices at each phase of stroke rehabilitation is essential [2].

Most bilateral-legged robots worldwide share a common therapeutic concept: providing symmetrical lower-limb movements that resemble normal gait patterns to facilitate neural mechanisms, such as the central pattern generator [2–7]. Despite this shared conceptual basis, emerging evidence suggests that responder characteristics vary by robotic mechanism. For example, individuals with higher trunk function benefit more from the Gait Trainer—an end-effector device [8]—while younger individuals who initiate RAGT earlier and undergo frequent training tend to achieve better outcomes with Lokomat, an exoskeleton-type device [9]. These findings suggest that even among devices with similar therapeutic goals, the optimal conditions for gait recovery are device-specific.

Differences in robotic mechanisms can influence the characteristics of patients who respond more favorably to each device. Therefore, the optimal conditions for promoting gait recovery may vary depending on the type of robotic assistance employed [7].

**Fig. 1:**
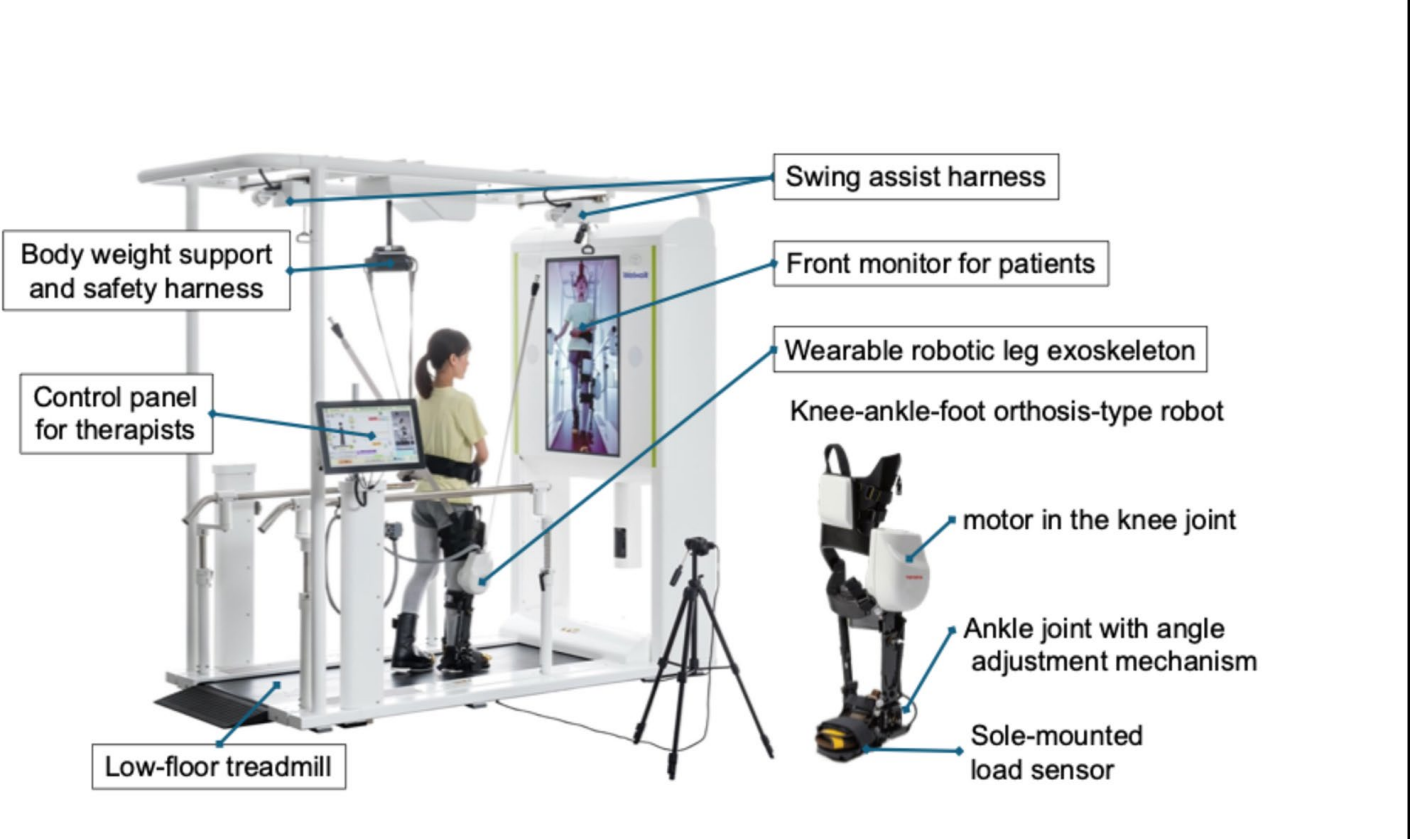
Overview of the Welwalk WW-2000. The Welwalk WW-2000 comprises a wearable robotic leg exoskeleton (knee-ankle-foot orthosis-type robot), low-floor treadmill, body weight support and safety harness, swing assist harness (which can be used as a robot weight-support device), front monitor for patient use, and control panel for the therapist. Specifically, the robotic leg is equipped with a motor in the knee joint and a sole-mounted load sensor, which is used to calculate the gait cycle. The robotic leg was worn only on the affected lower limb, and all robot operations were controlled using the therapist’s control panel.

Recently, the gait-training assistance robot Welwalk (Fig. 1)—designed to be worn on a single affected leg while enabling independent movement of both lower limbs—has been developed and is commercially available [10]. Welwalk—designed based on motor learning theory—is adaptable to various gait patterns and facilitates a walking practice that incorporates error-driven learning and compensatory strategies. Early studies suggested that both the developmental model (Gait Exercise Assist Robot [GEAR]) and commercialized Welwalk promote efficient gait independence [10–13]; however, a recent multicenter randomized controlled trial (RCT) [14] reported contrasting results. While the abovementioned RCT found no significant overall advantage of adding Welwalk to conventional physical therapy, it revealed that a subgroup of patients with cerebral infarction achieved gait independence significantly earlier. This discrepancy suggests that the benefits of Welwalk are not uniform across all patient profiles. Identifying the characteristics of individuals who best respond to this specific RAGT is crucial, particularly since research in this area is limited [15, 16].

Therefore, we aimed to identify the characteristics of best responders among non-ambulatory patients with severe hemiparesis in the subacute phase of stroke who underwent gait training with Welwalk, a single-legged gait-training assistance robot designed based on motor learning theory.

## Methods

### Study aim

In this study, we identified the characteristics of best responders among non-ambulatory individuals with severe hemiparesis in the subacute phase of stroke who underwent gait training with the Welwalk robotic device.

### Study design and setting

This retrospective observational cohort study was conducted at Okayama Rehabilitation Hospital, Japan, and adhered to the Strengthening the Reporting of Observational Studies in Epidemiology guideline [17]. The study was approved by the Ethics Committee of Okayama Rehabilitation Hospital (approval number: OR R6-2) and registered with the University Hospital Medical Information Network Clinical Trials Registry (UMIN-CTR, ID: UMIN000056551). The requirement for informed consent was waived due to the study’s retrospective nature; instead, an opt-out policy was used for patient inclusion.

### Study participants

This study was an exploratory, retrospective analysis of consecutive patients who underwent RAGT with Welwalk at our rehabilitation hospital. The study period spanned from February 2018—when Welwalk was first introduced at our institution—to September 2024.

Inclusion criteria included first-ever supratentorial stroke, a total score of ≤ 5 in the lower limb motor function items of the Stroke Impairment Assessment Set (SIAS) [18], and the inability to walk with supervision at the initiation of Welwalk training. Participants were excluded if they had subarachnoid hemorrhage, presented with neurological comorbidities other than stroke, declined participation, were unable to complete post-intervention assessments due to medical deterioration or hospital transfer, or underwent < 10 walking training sessions with Welwalk. Among the enrolled participants, 10 were included in a previous study [14] (UMIN-CTR clinical trial: UMIN000031959).

### Multidisciplinary rehabilitation including robot-assisted gait training

The participants received a combination of gait training using lower-limb orthoses and a walking-support robot. RAGT was performed using the Welwalk series (WW-1000 and WW-2000; Toyota Motor Corporation, Aichi, Japan). The Welwalk system comprises a wearable robotic leg exoskeleton (knee-ankle-foot orthosis-type robot), low-floor treadmill, body-weight support and safety harness, swing assist harness, front monitor for patients, and control panel for therapists (Fig. 1) [13, 14, 19]. The exoskeleton features a motorized knee joint, an adjustable ankle joint, and a sole-mounted load sensor. Its defining mechanical feature is its unilateral design: the robot attaches only to the affected limb, leaving the unaffected limb and trunk entirely free. The device’s weight is fully supported by a swing assist harness, allowing the user to walk freely and naturally. This configuration encourages engagement of motor functions for balance and propulsion, minimizes interference with natural gait, and permits functional compensatory strategies. The system’s control strategy is based on motor learning theory. Its “assist-as-needed” approach [20] respects the user’s intent and facilitates error-driven learning rather than enforcing a rigid, predetermined gait pattern. The system adapts to the patient’s variable movements in real time. Sole-mounted load sensors detect the gait cycle and trigger phase-dependent assistance to the motorized knee joint. Therapists can precisely customize the timing and magnitude of support for knee extension and flexion during the stance and swing phases, respectively. This customization allows therapists to provide the minimal assistance required to support movement of the affected limb, thereby encouraging maximal voluntary effort. The front monitor provides visual feedback through mirror or side-view whole-body images, along with top-down views of the feet. Audio feedback enhances participants’ understanding of critical events, such as knee emergence, episodes of giving way, and successful weight loading. The WW-2000 model features a front camera with a three-dimensional sensor and an additional capability to analyze gait by identifying irregular walking patterns [19]. However, the fundamental design of both models is nearly identical, with no difference in gait-support function.

Gait training using the Welwalk system aimed to enhance walking independence during rehabilitation. RAGT was initiated at the earliest appropriate time after hospitalization, following eligibility confirmation and consultation with a physician and therapist trained in Welwalk operation and training guidelines. Welwalk training was conducted 5–6 times per week in 40-min sessions. The intervention was delivered for at least 2 weeks by a physical therapist experienced with the Welwalk system. The RAGT period was initially set at 4 weeks but was shortened if participants achieved independent walking on flat ground without therapist assistance.

A rehabilitation program was provided for up to 180 min/day, including 60–80 min/day of physical therapy and 100–120 min/day of occupational and speech therapies. Multidisciplinary rehabilitation was tailored to individual needs, with the treatment plan and content determined through discussion between the attending physician and therapist.

In addition to RAGT using the Welwalk system, physical therapy included conventional interventions such as gait training with orthotics, joint range-of-motion exercises, muscle strengthening, functional movement training, and activities of daily living (ADL). Electrical and vibration stimulation devices were also used as needed.

### Clinical outcomes

Demographic, clinical, and functional parameters were retrospectively obtained from electronic medical records and Welwalk operation logs at the initiation and completion of the 4-week Welwalk training period. At the initiation of Welwalk training, we recorded the following data: (1) age; (2) sex; (3) stroke type; (4) affected side; (5) days to RAGT initiation (from stroke onset); (6) Gait Ability Assessment for hemiplegics (GAA) [21]; (7) cognitive sub-score of the Functional Independence Measure (FIM) [22]; and (8) lower-limb motor, lower-limb sensory, and trunk functions evaluated using the SIAS [18]. Data on the GAA score were obtained at the end of the 4-week training period. The assigned therapist assessed the GAA and SIAS weekly, while the assigned therapist and nurse evaluated the FIM weekly. Specifically, the primary outcome was defined as a binary variable indicating whether participants achieved walking with supervision or better (GAA score ≥ 5) at the end of the 4-week RAGT period. A GAA score ≥ 5 indicates the ability to walk with a short-leg orthosis and a cane without physical assistance from a therapist, although supervision may still be required for safety or guidance. The GAA is a walking ability assessment method designed to capture changes in walking independence during gait training by referencing the FIM scoring criteria [21].

Explanatory variables included the following nominal variables: sex (male or female), stroke type (cerebral hemorrhage or infarction), and affected side (left or right). Continuous and ordinal variables included age, days to RAGT initiation (defined as days from stroke onset to RAGT initiation), GAA score, FIM cognitive items (comprehension, expression, social interaction, problem solving, and memory), and SIAS subscores for lower-limb motor function (hip flexion, knee extension, and ankle dorsiflexion), sensory function (touch and position sense), and trunk function (verticality and abdominal muscles). Each continuous and ordinal variable was dichotomized at the median for further analysis. The FIM was developed to comprehensively assess ADL impairment, comprising sub-items for self-care, elimination management, transfers, communication, and social cognition. Specifically, the scale comprises a 13-item motor and 5-item cognitive subscale. This functional performance scale is widely used in clinical practice and has been evaluated for interrater reliability and validity [23, 24]. Each item is scored on a scale from 1 (complete dependence) to 7 (complete independence). The SIAS is an overall index of stroke-induced functional impairment that has been examined for reliability and validity [25]. In this study, SIAS was used to assess the motor and sensory functions of the affected leg, as well as trunk functions.

### Statistical Analysis

We used Direct Linear Non-Gaussian Acyclic Model (Direct-LiNGAM), a powerful tool for causal discovery and variable selection, for analysis [26]. Direct-LiNGAM assumes that the observed data are generated from a linear, non-Gaussian structural equation model with no unobserved confounders, providing improved accuracy and computational efficiency compared with traditional methods [27]. This method estimates the causal ordering of variables by iteratively identifying the most exogenous variable, based on the independence between external influences and residuals. Given these considerations, Direct-LiNGAM was used to identify variables exerting direct causal effects on the primary outcome. The analysis included variables such as age; sex; stroke type; affected side; days to RAGT initiation; GAA scores; cognitive sub-score of the FIM; and lower-limb motor, lower-limb sensory, and trunk functions assessed by the SIAS. These variables were selected based on their relevance to the primary outcome and previous studies in the field [15, 16].

A bootstrap procedure with 1,000 iterations was implemented to ensure the robustness of the causal structure estimation. In each iteration, a bootstrap sample was created through random sampling with replacement, and Direct-LiNGAM was fitted to this sample. From each fitted model, an adjacency matrix representing the causal relationships was extracted, and only causal effects with absolute values exceeding 0.05 were retained.

The final causal structure was determined through several steps. First, the causal effect strengths were averaged across all bootstrap iterations. Only the direction with the strongest causal effect was retained in cases where bidirectional relationships were identified. Subsequently, the resulting causal graph for cycles were examined using the cycle-detection algorithm of the NetworkX library. Cycles found in the graph were resolved by iteratively removing the weakest edge until an acyclic graph was obtained. This entire analysis was implemented using Python with the “lingam” package (https://lingam.readthedocs.io/).

Logistic regression analysis was conducted using the variables selected by Direct-LiNGAM to examine their association with achieving walking with supervision (GAA score ≥ 5) at 4 weeks. Odds ratios (ORs) with 95% confidence intervals (CIs) were calculated, and statistical significance was set at *p* < 0.05.

Direct-LiNGAM was originally designed for linear regression with continuous outcomes; however, it was applied in this study to the binary outcome of walking with supervision achievement. The Least Absolute Shrinkage and Selection Operator (LASSO) logistic regression was performed to assess the robustness of variable selection. All numerical variables were standardized, and categorical variables were dummy-coded prior to analysis. The penalty parameter (λ) was determined using five-fold cross-validation, which was employed solely to tune the regularization strength and reduce overfitting, thereby improving internal validity without providing external validation. Importantly, LASSO was applied only as a variable selection method to evaluate the robustness of predictors identified by Direct-LiNGAM, and not for causal inference. The Linear non-Gaussian model for Mixed data (LiM), an extension of Direct-LiNGAM that accommodates both continuous and discrete variables, was applied as an additional sensitivity analysis [28]. A total of 1,000 bootstrap replications were generated. For each directed edge, we computed the mean causal weight and a stability measure—defined as the proportion of bootstrap samples in which the edge appeared with the same sign. We retained only edges with an absolute mean weight > 0.05 and a stability measure ≥ 0.20 (i.e., present in at least 20% of bootstrap samples) for reporting. We considered leave-one-out or split-sample validation; however, because causal graph estimation is sensitive to small data perturbations, data splitting may increase structural variability and may not provide reliable validation. Therefore, we evaluated robustness using sensitivity analyses and bootstrap-based stability assessment instead.

All statistical analyses were performed using Python version 3.11.7. Direct-LiNGAM and LiM analyses were implemented using the “lingam” package, while logistic regression with LASSO regularization was performed using the LogisticRegression function (L1 penalty) from scikit-learn (sklearn.linear_model).

## Results

### Study Flow

**Fig. 2:**
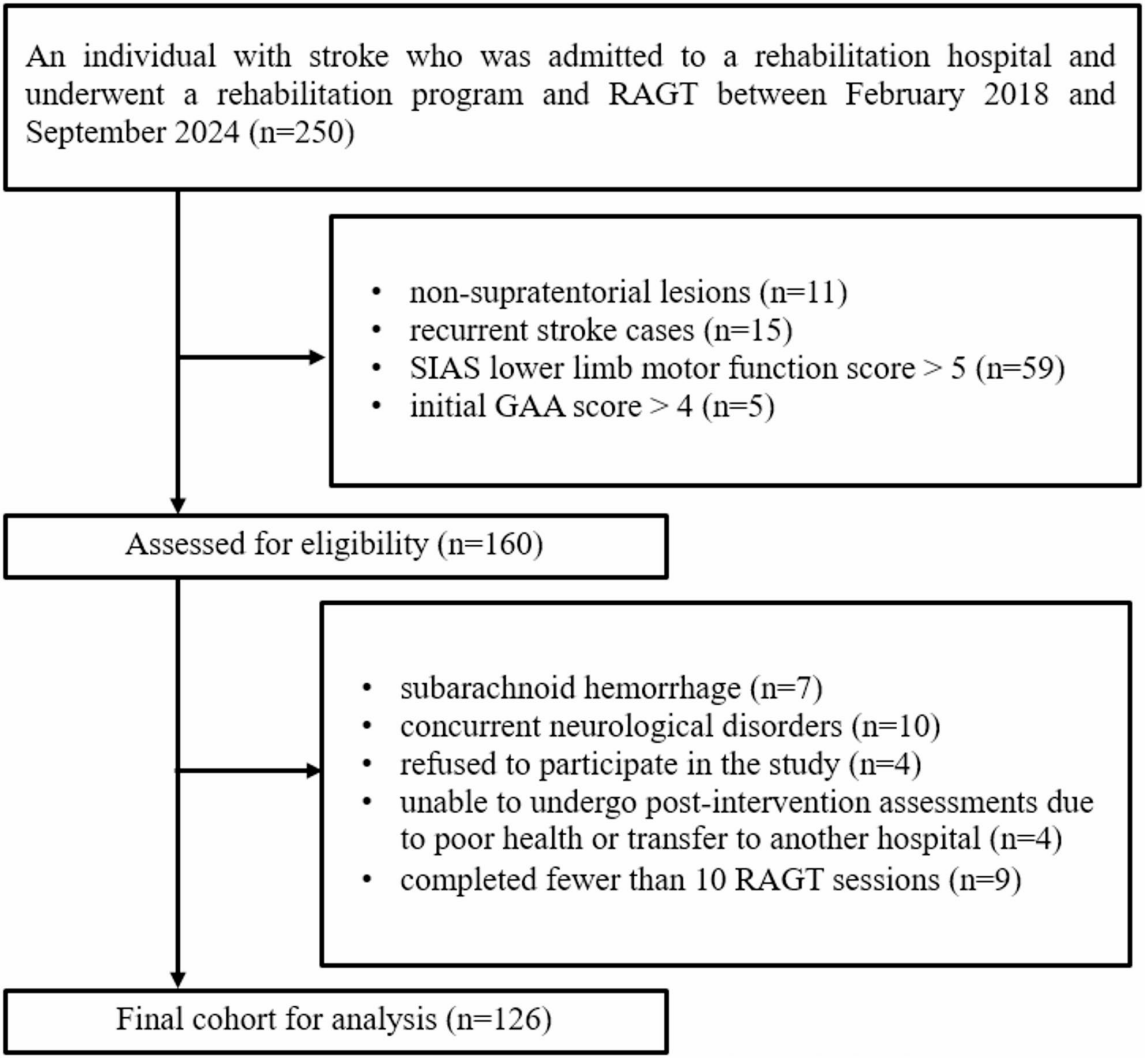
Flow diagram of participant selection. A total of 126 participants were included in the final analysis after applying all exclusion criteria. RAGT, robot-assisted gait training; SIAS, Stroke Impairment Assessment Set; GAA, Gait Ability Assessment

Figure 2 shows the participant selection process. A total of 250 participants were initially screened for eligibility between February 2018 and September 2024. Participants were excluded based on the following criteria: non-supratentorial lesions (*n* = 11), recurrent stroke (*n* = 15), SIAS score for lower-limb motor function > 5 (*n* = 59), and GAA score > 4 (*n* = 5). Individuals with subarachnoid hemorrhage (*n* = 7), presenting with concomitant neurological disease (*n* = 10), who declined participation in the study (*n* = 4), unable to perform post-intervention assessments due to ill health or hospital transfer (*n* = 4), or those who completed < 10 RAGT sessions (*n* = 9) were also excluded. Ultimately, 126 participants were included in the final analysis. All data were complete, with no missing values.

### Baseline characteristics

The mean age of the participants was 61.2 years (standard deviation = 11.8), and 29.4% (*n* = 37) were female. Ischemic stroke accounted for 37.3% (*n* = 47) of cases, and 42.9% (*n* = 54) had left-sided hemiplegia. The median time from stroke onset to RAGT initiation and the baseline GAA score was 42.5 days (interquartile range [IQR]: 34.0‒54.8) and 3.0 points [IQR: 2.0‒3.0], respectively. Table 1 presents the detailed baseline characteristics.

**Table 1:** Baseline characteristics of participants grouped by walking status at 4 weeks

*n*	Overall	Walking with physical assistance	Walking with supervision
	126	60	66
Age, years	61.2 (11.8)	62.3 (12.0)	60.1 (11.7)
Sex, female, n (%)	37 (29.4)	16 (26.7)	21 (31.8)
Stroke type, infarction, n (%)	47 (37.3)	21 (35.0)	26 (39.4)
Affected side, Left side, n (%)	54 (42.9)	28 (46.7)	26 (39.4)
Days to RAGT initiation	42.5 [34.0, 54.8]	52.0 [40.0, 60.0]	37.0 [28.0, 48.0]
GAA	3.0 [2.0, 3.0]	2.0 [2.0, 3.0]	3.0 [3.0, 4.0]
SIAS			
Hip flexion	0.5 [0.0, 1.0]	0.0 [0.0, 1.0]	1.0 [0.0, 2.0]
Knee extension	0.0 [0.0, 1.0]	0.0 [0.0, 0.0]	0.5 [0.0, 1.8]
Ankle dorsiflexion	0.0 [0.0, 0.0]	0.0 [0.0, 0.0]	0.0 [0.0, 0.0]
Touch sensation	1.0 [0.0, 2.0]	1.0 [0.0, 2.0]	2.0 [1.0, 2.0]
Joint position sense	1.0 [0.0, 2.0]	0.0 [0.0, 1.0]	1.0 [0.0, 2.0]
Trunk verticality	3.0 [2.0, 3.0]	2.0 [1.0, 3.0]	3.0 [3.0, 3.0]
Abdominal muscle strength	1.0 [0.0, 2.0]	1.0 [0.0, 1.0]	2.0 [1.0, 2.0]
FIM			
Cognitive Score	19.0 [12.2, 23.0]	15.5 [10.0, 20.2]	21.0 [16.0, 25.0]
Comprehension	4.0 [3.0, 5.0]	3.0 [2.0, 5.0]	4.0 [3.0, 5.0]
Expression	4.0 [2.0, 5.0]	3.0 [2.0, 5.0]	4.0 [3.0, 5.0]
Social interaction	4.5 [3.0, 5.0]	4.0 [2.0, 5.0]	5.0 [4.0, 6.0]
Problem-solving	3.0 [2.0, 4.8]	2.0 [1.0, 4.0]	4.0 [2.2, 5.0]
Memory	3.0 [2.0, 5.0]	2.0 [1.0, 3.0]	3.0 [3.0, 5.0]

Values are presented as mean (standard deviation), median [interquartile range], or n (%), as appropriate. For ordinal variables, medians are displayed with one decimal place for consistency. Sex and stroke type indicate the number of females and cerebral infarction cases, respectively. The affected side represents the number of individuals with left-sided hemiplegia, and days to RAGT initiation denote the number of days from stroke onset to RAGT initiation. Depending on the item, the SIAS scores range from 0 (severe impairment) to 3‒5 (no impairment). The FIM cognitive scores ranged from 1 (complete dependence) to 7 (complete independence) for each item

SIAS, Stroke Impairment Assessment Set; FIM, Functional Independence Measure; GAA, Gait Ability Assessment; RAGT, robot-assisted gait training

### Causal structure analysis

**Fig. 3:**
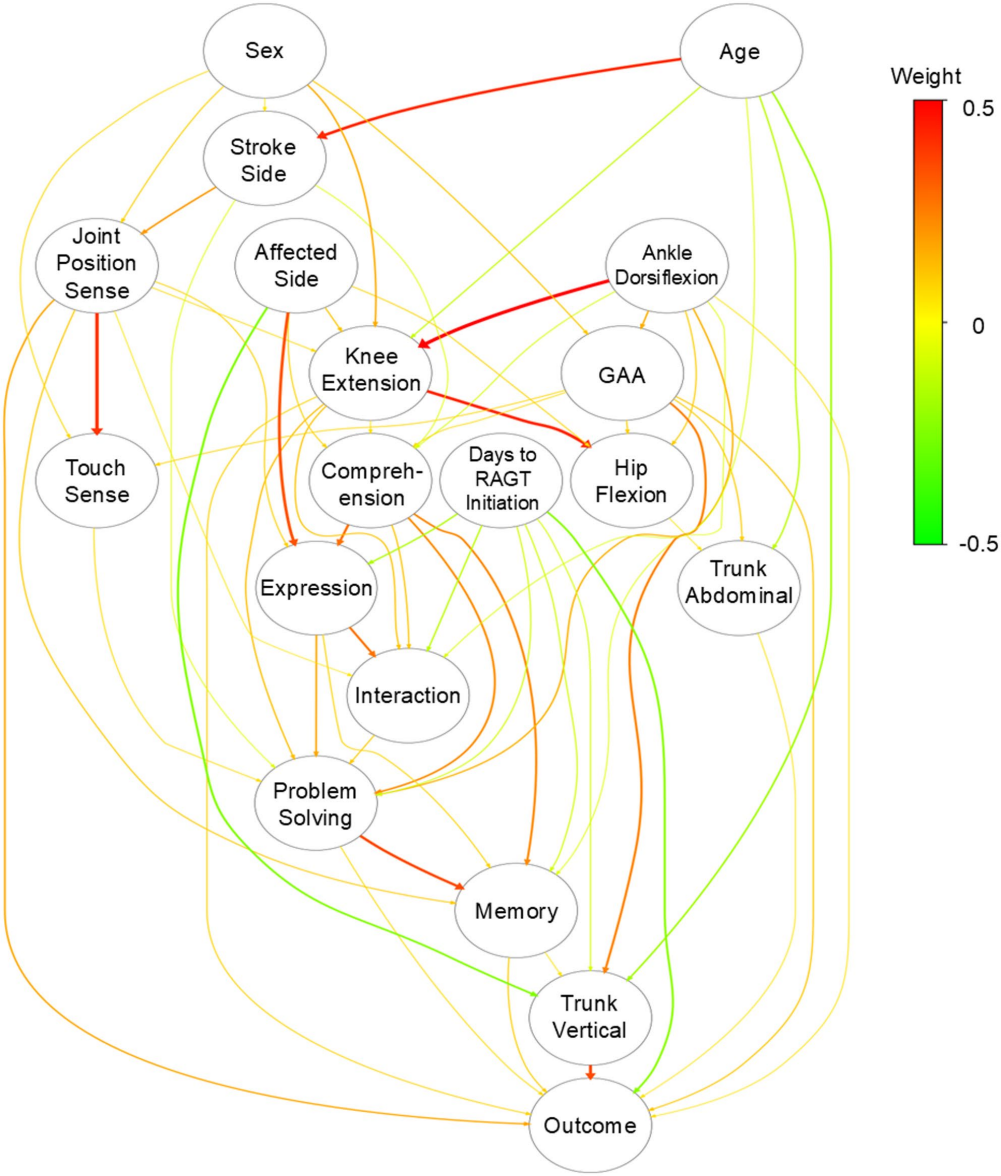
Causal structure identified by Direct-LiNGAM analysis. Directed graph showing causal relationships among variables identified by Direct-LiNGAM analysis. Arrows indicate the direction of causal effects. The outcome variable (achieving walking with supervision) was causally influenced by multiple variables, with the strongest direct effects observed for trunk verticality, days to RAGT initiation (days from stroke onset to RAGT initiation), and joint position sense. For clarity, only causal effects with absolute values > 0.05 are displayed. Variables without direct causal paths to the outcome were excluded from subsequent analyses. The color scale indicates edge weights (direct causal effects) ranging from − 0.5 to 0.5. Edge color represents both the direction and magnitude of the causal effect, with red indicating stronger positive effects, green indicating stronger negative effects, and yellow indicating values near zero. Edge thickness is proportional to the absolute value of the causal effect. Direct-LiNGAM, Direct Linear Non-Gaussian Acyclic Model; SIAS, Stroke Impairment Assessment Set; RAGT, robot-assisted gait training; GAA, Gait Ability Assessment; FIM, Functional Independence Measure

Direct-LiNGAM analysis identified the following nine variables with direct causal effects on walking with supervision achievement (Fig. 3): knee extension, ankle dorsiflexion, joint position sense, trunk verticality, abdominal muscle strength, problem-solving, memory, days to RAGT initiation, and initial GAA score.

### Primary analysis

**Fig. 4:**
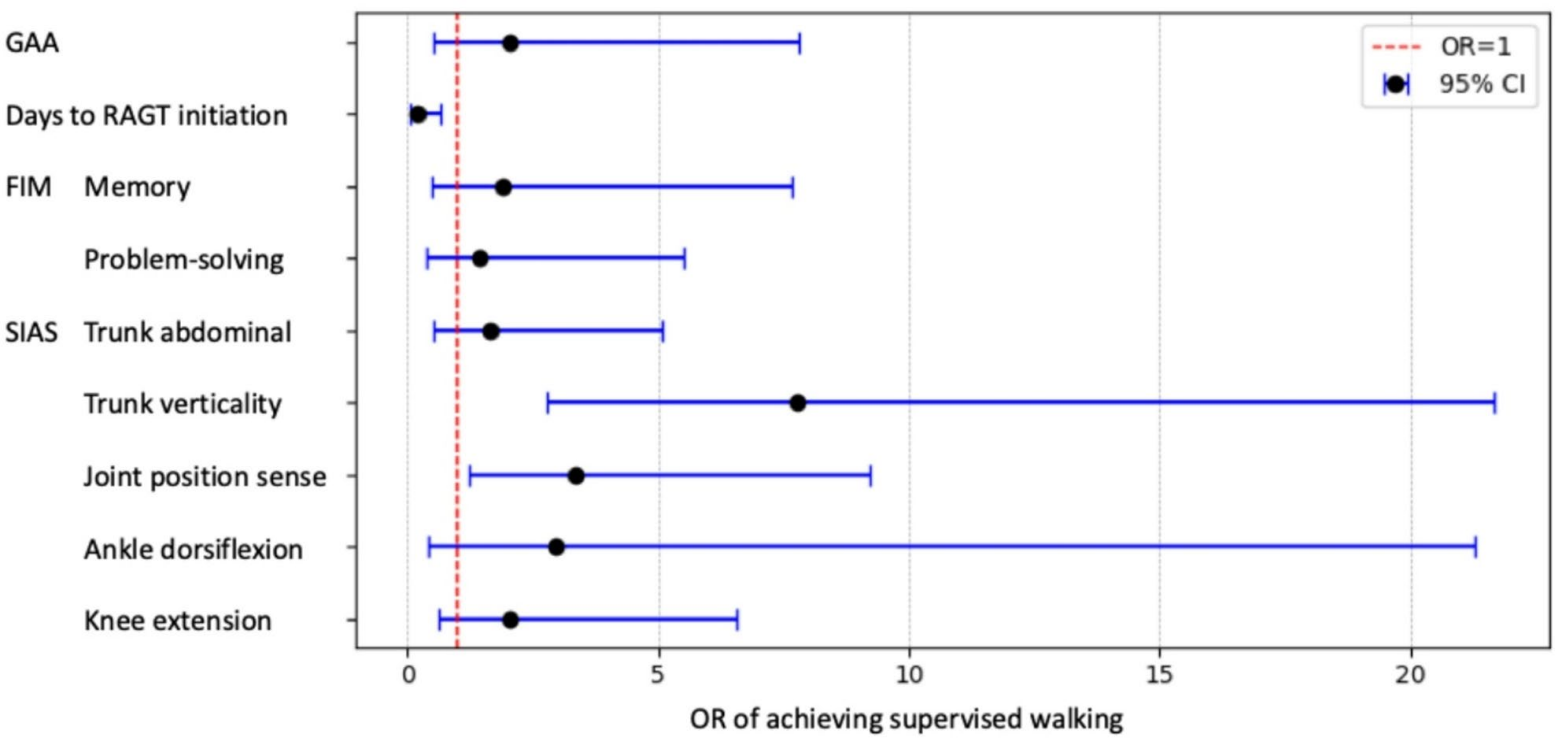
Forest plot of odds ratios (ORs) for achieving walking with supervision at 4 weeks. The ORs with 95% confidence intervals (CIs) from the logistic regression analysis. Vertical dashed line represents OR = 1 (no effect). Points represent the estimated ORs, and the horizontal lines represent 95% CIs. Significant associations (*p* < 0.05) were observed for trunk verticality (OR = 7.79, 95% CI: 2.80‒21.68, *p* = 0.0001), joint position sense (OR = 3.37, 95% CI: 1.23‒9.24, *p* = 0.0183), and days to RAGT initiation (days from stroke onset to RAGT initiation) (OR = 0.19, 95% CI: 0.06‒0.66, *p* = 0.0088). SIAS, Stroke Impairment Assessment Set; RAGT, robot-assisted gait training; GAA, Gait Ability Assessment; FIM, Functional Independence Measure

Logistic regression identified three variables significantly associated with achieving walking with supervision (Fig. 4). Higher trunk verticality (OR = 7.79; 95% CI: 2.80–21.68, *p* = 0.0001) and preserved joint position sense (OR = 3.37; 95% CI: 1.23–9.24, *p* = 0.0183) were associated with an increased likelihood of success, whereas a longer time to RAGT initiation was associated with a decreased likelihood (OR = 0.19; 95% CI: 0.06–0.66, *p* = 0.0088).

### Robustness of variable selection

**Fig. 5:**
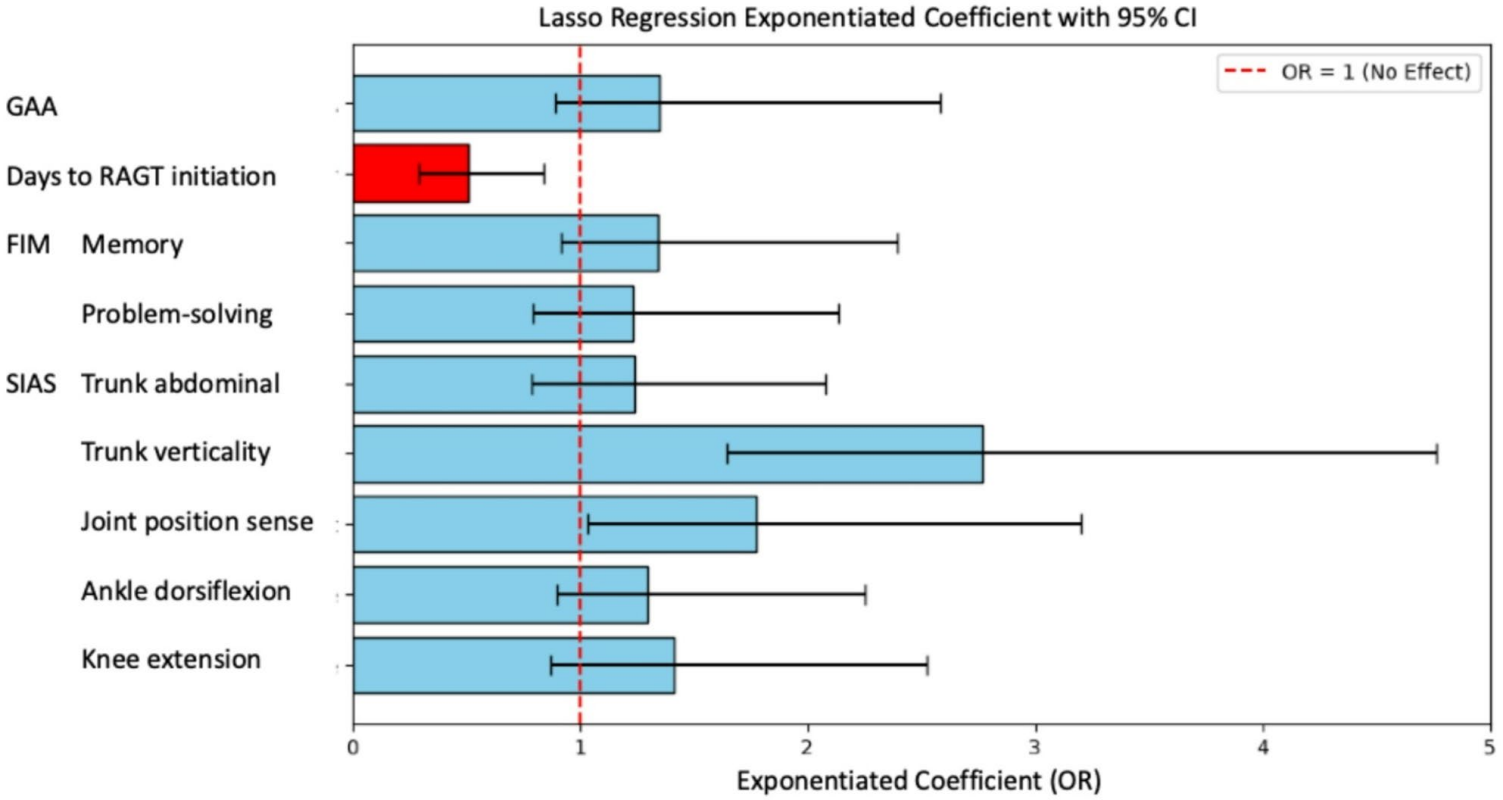
Forest plot of LASSO regression results for the validation of variable selection. Exponentiated coefficients [equivalent to odds ratios (ORs)] with 95% confidence intervals (CIs) from the LASSO regression analysis. The vertical dashed line represents OR = 1 (no effect). Bars represent 95% CIs, with blue and red indicating positive and negative associations, respectively. Trunk verticality (OR = 2.77, 95% CI: 1.65‒4.76), joint position sense (OR = 1.78, 95% CI: 1.04‒3.21), and days to RAGT initiation (days from stroke onset to RAGT initiation) (OR = 0.51, 95% CI: 0.30‒0.84) showed significant associations consistent with the primary analysis results. LASSO, Least Absolute Shrinkage and Selection Operator; GAA, Gait Ability Assessment; SIAS, Stroke Impairment Assessment Set; RAGT, robot-assisted gait training; FIM, Functional Independence Measure

LASSO regression analysis showed similar patterns (Fig. 5), with estimated ORs for trunk verticality (OR = 2.77; 95% CI: 1.65‒4.76), joint position sense (OR = 1.78; 95% CI: 1.04‒3.21), and days to RAGT initiation (OR = 0.51; 95% CI: 0.30‒0.84).

In the LiM analysis (see Supplementary Fig. 1, Additional File 1), days to RAGT initiation and trunk verticality reemerged as direct determinants of achieving walking with supervision, consistent with the findings from the main analysis. Joint position sense did not show a direct edge; instead, the LiM indicated a pathway from tactile sensation to joint position sense.

## Discussion

This study was based on a retrospective cohort of individuals with severe motor paralysis who required ambulatory assistance due to a first-ever subacute stroke and underwent a multidisciplinary program at a rehabilitation unit in Japan. After a 4-week Welwalk gait training program, we identified three significant predictors of achieving walking with supervision in individuals with subacute stroke and severe hemiparesis: earlier initiation of RAGT (OR = 0.19), trunk verticality (OR = 7.79), and lower-limb joint position sense (OR = 3.37). Therefore, the predictive model suggested that the best responders were individuals who initiated RAGT early and demonstrated preserved trunk stability and proprioception.

Early initiation of RAGT using the Lokomat [9] has been associated with a time window of functional recovery. A Cochrane review recommends implementing RAGT within 3 months after onset [1], and the findings of the present study support this recommendation. In this study, because all participants received RAGT within 3 months post-stroke, we could not fully evaluate the optimal timing within this period. Nevertheless, responders initiated RAGT significantly earlier (median: 37 days from onset) than non-responders (median: 52 days from onset). These findings suggest that initiating RAGT within approximately 1–2 months after onset may provide greater benefit than the broader 3-month window recommended in the Cochrane review.

In Welwalk training, better trunk verticality was associated with a higher likelihood of achieving walking with supervision. A previous study using the Gait Trainer indicated that the benefits of RAGT similarly diminish in the absence of preserved trunk function [8]. As an end-effector-type device, the Gait Trainer permits free hip joint movement and provides partial trunk weight support without full fixation, enabling users to practice trunk control. Similarly, the Welwalk system allows users to train with free hip and trunk movement, which may explain the consistent findings between systems. Studies investigating best responders using the GEAR, a prototype of Welwalk, demonstrated that trunk verticality—measured using the SIAS—was associated with achieving walking with supervision [15]. Collectively, these findings underscore the critical role of trunk function in gait recovery when using robotic systems that permit hip joint degrees of freedom.

A notable finding of this study pertains to the proprioceptive sensation domain of the SIAS. Previous studies have shown that independent walking is possible if the knee extensor muscle strength on the affected side is adequate, even in the presence of impaired proprioceptive sensation [29]. In this study, the median knee extension score at baseline, measured by the SIAS, was 0 (Table 1), indicating that the participants had insufficient muscle strength. Therefore, proprioceptive sensation may play a crucial role in compensating for lower-limb muscle weakness through motor control by ensuring single-leg support on the affected side, which is necessary for walking. Joint position sense did not show a direct edge to the outcome in the LiM sensitivity analysis; instead, it received an incoming path from tactile sensation. This finding suggests that joint position sense is not an entirely independent determinant, but that sensory function as a whole—including proprioception and tactile sensation—contributes to gait acquisition. Therefore, while the predictive model highlighted joint position sense, the result should be interpreted cautiously and considered within the broader context of sensory function.

Patients with severe hemiplegia typically have limited functional recovery [30], and the use of “compensation” that deviates from normal movement patterns is considered an important strategy for overcoming challenges [31–33]. Such patients reportedly tend to rely on compensation strategies involving multiple joints, including trunk movements, to compensate for lost motor functions [33–35].

Bilateral-legged robots can reproduce gait patterns similar to those of normal gait by controlling both lower limbs’ movement trajectories, even in the presence of paralysis. However, the degrees of freedom in movement are limited. Single-legged robots partly control only the affected lower limb, leveraging the remaining freedom in movement to flexibly adapt to individual patients’ compensatory walking patterns.

The Welwalk used in this study is a unilaterally applied exoskeleton designed to regulate the knee and ankle joints while preserving the mobility of the compromised hip joint, unaffected lower limb, and the trunk. In gait rehabilitation using Welwalk for patients with severe hemiplegia, we propose that restoring weight-bearing capacity during the stance phase is a crucial factor—that is, the recovery of stability. Welwalk’s active knee assistance, synchronized with the patient’s gait cycle, intelligently addresses the conflicting dynamic challenges of ensuring stability during the stance phase and natural mobility during the swing phase, even with inconsistent gait rhythms [19, 36]. Building upon this stability, the system encourages the patient to actively generate the propulsive force necessary for walking using their residual function (particularly the muscles around the hip joint, trunk, and unaffected lower limb). Rather than compensating for all joint movements required for normal walking, this treatment approach promotes more effective recovery by maximizing the patient’s active participation within a minimally supportive environment necessary for stability and walking establishment. This structural feature suggests the potential to provide a training environment that promotes the learning of compensation strategies tailored to individual patient abilities. We believe that joint position sense and trunk verticality—identified as key factors among the best responders—contribute to the effectiveness of a training environment that facilitates the acquisition of compensation strategies tailored to each patient’s individual capabilities.

A recent study of the best responders to Welwalk reported an association between the efficiency of walking improvement during RAGT and the total FIM cognitive function score [16], suggesting that cognitive function influences efficient motor learning. However, in the present study, the total FIM cognitive function score was not included in the analysis to examine the specific involvement of cognitive function. FIM scores for memory and problem-solving were selected as potential factors; however, no significant differences were observed. The median total FIM cognitive function score for participants was 23.0 (20.0, 28.0) in a previous study [16] compared with 19.0 (12.2, 23.0) in this study. Differences in baseline cognitive function, outcome (improvement efficiency in walking independence and presence/absence of walking with supervision), and RAGT duration may have contributed to the inconsistent trends observed in this study compared with those reported in the previous study [16]. A systematic review of independent walking in patients with stroke [37] also emphasizes the importance of cognitive function and highlights the need for further research in this field.

Traditional variable selection methods, such as LASSO regression, focus on optimization while considering the causal relationships among variables. However, LiNGAM enables the explicit modeling of causal structures among variables. In this study, applying LiNGAM facilitated the identification of causal relationships between the factors influencing gait recovery. The variable selection results obtained through LiNGAM were supported by LASSO validation. Therefore, the importance of the identified factors in this study was confirmed using causal and predictive approaches. We gained new insights from a causal perspective, particularly highlighting the importance of early intervention, trunk function, and proprioception. Our approach using LiNGAM demonstrates the potential to advance the understanding of causal mechanisms in rehabilitation medicine, extending beyond mere predictive model construction. This methodology could contribute to the development of more targeted and effective rehabilitation strategies based on causal relationships rather than mere statistical associations.

This study has some limitations. First, as a single-center retrospective study, its external validity is inherently limited. Patients eligible for RAGT may not have been admitted to the study facility, and individuals with stroke types other than severe hemiplegia or those with extrahemispheric lesions may have exhibited different response patterns. We applied five-fold cross-validation within the LASSO procedure to tune the penalty parameter and reduce the risk of overfitting; however, this approach only improves internal validity and does not ensure external validity. Therefore, the generalizability of our findings remains limited by the study design. Second, while the number and duration of training sessions were controlled, their content and intensity were unstandardized. Programs were individualized based on each patient’s condition and goals, which may have introduced variability. Moreover, standardizing training protocols given the heterogeneity in patients with severe subacute stroke remains a challenge for future research. Third, we evaluated outcomes after a 4-week training period. Consequently, longer interventions might yield different or more significant effects on functional recovery. Fourth, in the absence of a control group, the identified predictors may primarily reflect general prognostic factors for gait recovery in patients with severe hemiplegia. Nevertheless, we posit that their relevance is accentuated by the therapeutic mechanisms inherent in our single-legged RAGT. The optimal use of the device necessitates active patient engagement, with trunk verticality constituting a prerequisite for balance and sensory function being indispensable for the ‘assist-as-needed’ feedback loop. Therefore, these findings contribute to delineating the patient profile that is most appropriately matched to the specific demands of this technology, pending validation in a comparative trial.

Finally, Direct-LiNGAM was applied to a binary outcome, despite its original design for continuous variables. This approach represents a methodological compromise; however, applications including binary variables have been reported in the literature [38]. Direct-LiNGAM was used strictly for structure-aware variable screening in our study, while associations with the binary outcome were quantified by logistic regression. Moreover, a sensitivity analysis using LiM, a mixed-data extension of Direct-LiNGAM, reproduced key predictors (days to RAGT initiation and trunk verticality). However, we did not adopt LiM as the main analytic framework since it has not been established as a practical standard tool. Notably, LiM currently lacks widely accepted defaults for handling prior information, thresholding, and stability criteria, and its computational stability in small-to-moderate sample sizes remains limited. Therefore, we retained Direct-LiNGAM combined with logistic regression as the main analysis and presented LiM solely as a sensitivity analysis.

In summary, we identified key factors for achieving walking with supervision among patients with severe hemiplegia who underwent RAGT using a single-legged robot. While these results align with the original research objectives, they should be cautiously interpreted, given the abovementioned limitations. Furthermore, the lack of standardization in rehabilitation intensity may have introduced unmeasurable confounding factors. Comparisons with previous studies [8, 9, 15] support the association between trunk function and early intervention. Our new findings on the importance of joint position sense align with emerging evidence in neurorehabilitation, particularly in relation to motor learning and compensation strategies. Together, these findings contribute to the growing body of evidence aimed at optimizing individualized robot-assisted rehabilitation and emphasize the need for future prospective, multicenter collaborative studies to validate these causal pathways.

## Conclusions

In this study, we identified days to RAGT initiation, trunk verticality, and lower-limb joint position sense as key factors associated with early walking achievement among individuals with severe hemiplegia who underwent 4 weeks of RAGT using a single-legged robot. These findings highlight key factors for selecting individuals who may benefit from single-legged gait-training assistance robots and may help inform more effective intervention strategies.

## Supplementary Information

Below is the link to the electronic supplementary material.

Additional file 1: Title of data: Results of the Linear non-Gaussian model for Mixed data (LiM) analysis. Description of data: Directed edges represent estimated causal relationships obtained from 1,000 bootstrap replications. The numbers on each edge indicate mean causal weights, and only edges with absolute mean weights > 0.05 and stability measure ≥ 0.20 (i.e., present in at least 20% of bootstrap samples) were retained. Solid and dashed lines represent edges with absolute mean weights > 0.20 and ≤ 0.20, respectively. Days to RAGT initiation and trunk verticality showed direct connections to the outcome (walking with supervision), consistent with the main analyses. In contrast, joint position sense received an incoming edge from tactile sensation, and age also appeared only as a sink node, suggesting that their independent roles were less stable across methods and not consistently supported as direct determinants in this sensitivity analysis. Abbreviations: GAA, Gait Ability Assessment for Hemiplegics; RAGT, robot-assisted gait training.

## Data Availability

The datasets used and/or analyzed in this study are available from the corresponding author upon reasonable request.

## Abbreviations

RAGT: Robot-Assisted Gait Training
SIAS: Stroke Impairment Assessment Set
GAA: Gait Ability Assessment
FIM: Functional Independence Measure
Direct-LiNGAM: Direct Linear Non-Gaussian Acyclic Model
LASSO: Least Absolute Shrinkage and Selection Operator
UMIN-CTR: University Hospital Medical Information Network Clinical Trials Registry
IQR: Interquartile range
ORs: Odds ratios
CIs: Confidence intervals
ADL: Activities of daily living
GEAR: Gait Exercise Assist Robot
LiM: Linear non-Gaussian model for Mixed data
