# Supplementary material for: Key factors for successful gait acquisition in individuals with severe hemiplegic subacute stroke using a single-legged gait-training assistance robot: a retrospective cohort study
Source: J Neuroeng Rehabil. 2026 Mar 28;23:152. doi: 10.1186/s12984-026-01961-4 (PMC13151260; doi:10.1186/s12984-026-01961-4)
Supplement: Supplementary file 1 — Additional file 1: Title of data: Results of the Linear non-Gaussian model for Mixed data (LiM) analysis. Description of data: Directed edges represent estimated causal relationships obtained from 1,000 bootstrap replications. The numbers on each edge indicate mean causal weights, and only edges with absolute mean weights > 0.05 and stability measure ≥ 0.20 (i.e., present in at least 20% of bootstrap samples) were retained. Solid and dashed lines represent edges with absolute mean weights > 0.20 and ≤ 0.20, respectively. Days to RAGT initiation and trunk verticality showed direct connections to the outcome (walking with supervision), consistent with the main analyses. In contrast, joint position sense received an incoming edge from tactile sensation, and age also appeared only as a sink node, suggesting that their independent roles were less stable across methods and not consistently supported as direct determinants in this sensitivity analysis. Abbreviations: GAA, Gait Ability Assessment for Hemiplegics; RAGT, robot-assisted gait training. [file 12984_2026_1961_MOESM1_ESM.docx]

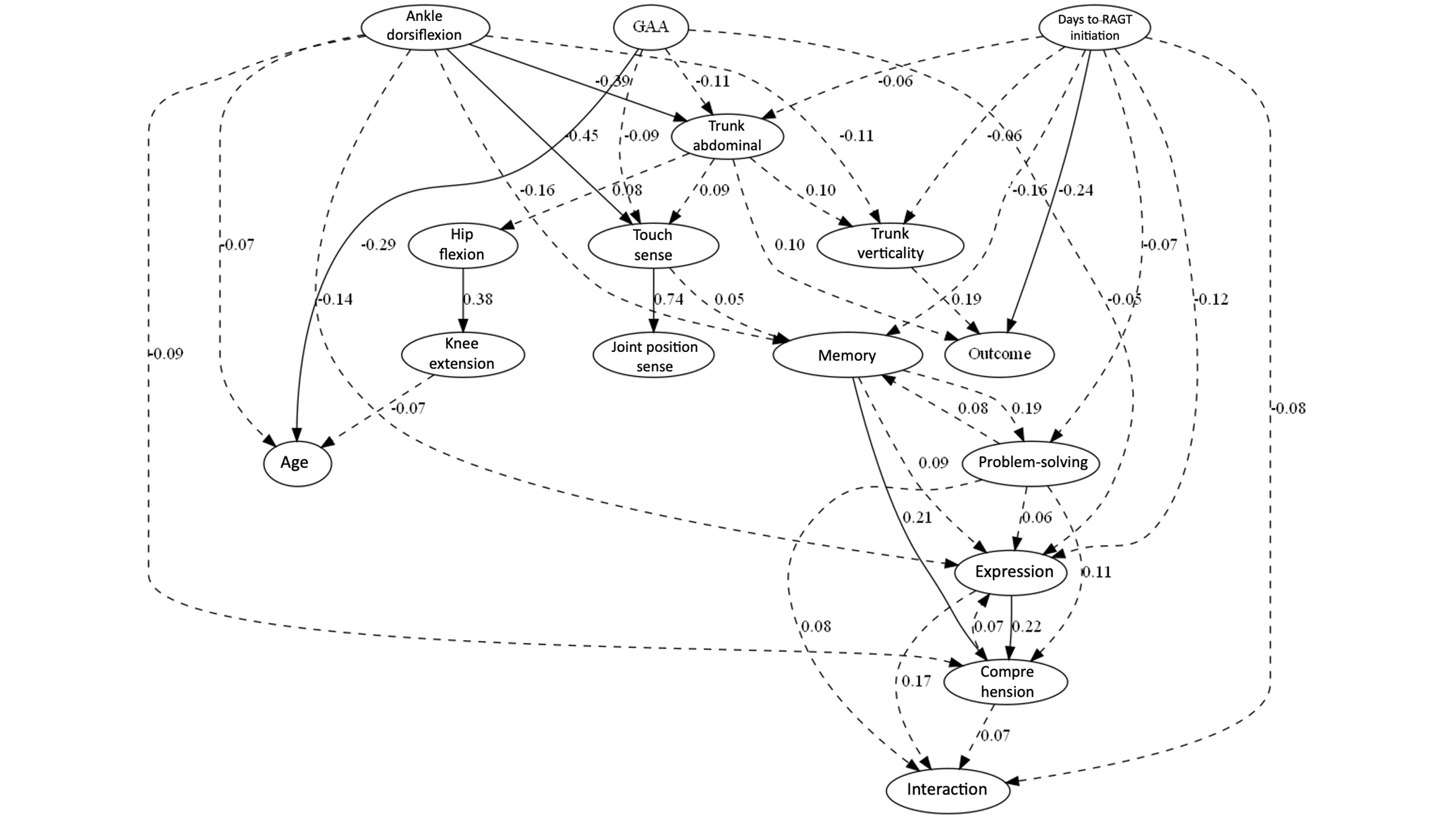


**Additional file 1**. Results of the Linear Non-Gaussian model for Mixed data (LiM) analysis.

Directed edges represent estimated causal relationships obtained from 1,000 bootstrap replications. The numbers on each edge indicate mean causal weights, and only edges with absolute mean weights >0.05 and stability measure ≥0.20 (i.e., present in at least 20% of bootstrap samples) were retained. Solid and dashed lines represent edges with absolute mean weights >0.20 and ≤0.20, respectively. Days to RAGT initiation and trunk verticality showed direct connections to the outcome (walking with supervision), consistent with the main analyses. In contrast, joint position sense received an incoming edge from tactile sensation, and age also appeared only as a sink node, suggesting that their independent roles were less stable across methods and not consistently supported as direct determinants in this sensitivity analysis.

Abbreviations: GAA, Gait Ability Assessment for Hemiplegics; RAGT, robot-assisted gait training.
